# Development of low cost ZnO based chemi resistive biosensor for the detection of vitamin B6 mediated by quantum dots

**DOI:** 10.1038/s41598-025-95892-0

**Published:** 2025-04-02

**Authors:** Bhavana Anchan, Saritha Kamath U, Suresh D. Kulkarni, Kavita A. Pandey, Shounak De, Ajeetkumar Patil

**Affiliations:** 1 Manipal Institute of Applied Physics (MIAP), Manipal Academy of Higher Education (MAHE), Manipal, 576 104 India; 2 Department of Medical Laboratory Technology, Manipal College of Health Professions, Manipal Academy of Higher Education (MAHE), Manipal, 576 104 India; 3 Centre for Nano and Soft Matter Sciences (CeNS), Arkavathi, Survey No.7, Shivanapura, Dasanapura Hobli, Bengaluru, 562162 India; 4 Department of Electronics and Communication Engineering, Manipal Institute of Technology (MIT), Manipal Academy of Higher Education (MAHE), Manipal, 576104 India

**Keywords:** Metal oxide thin film, Biosensor, Vitamin B molecules, Quantum dots, Nanoscale devices, Biosensors

## Abstract

In this study, we developed a chemi-resistive sensor for vitamin B6 mediated by CdTe quantum dots (Cadmium Telluride QDs) utilizing ZnO-based thin films through the spin-coating sol–gel technique annealed at 500 °C. The sensor successfully recognized vitamin B6 (Pyridoxine) in concentrations ranging from 2 to 10 µM by employing CdTe quantum dots (Cadmium Telluride QDs) on the ZnO thin film. This detection was achieved by implementing an amperometric (I–V) method, typically operating at a working voltage below 2 V. From the slope of the calibration curve, the sensitivity, and the limit of detection values are estimated to be 7.56 ± 0.92 nA/µM and 0.906 µM (n = 5), respectively. This study conducted electrochemical impedance spectroscopy (EIS) in conjunction with current–voltage (I–V) measurements. The results obtained from EIS are consistent with the current response observed in the I–V measurements. The sensing mechanism primarily relies on the charge transfer phenomenon (electrostatic attraction) between the analyte (vitamin B6 mediated by CdTe quantum dots) and the ZnO thin film. In summary, our work introduces a simple, rapid, and cost-effective ZnO thin film-based sensing device for detecting vitamin B6, mediated by CdTe QDs. This sensor holds promise for a wide range of applications, from pharmaceutical quality control to point-of-care diagnostics and routine vitamin B screening.

## Introduction

Vitamins mainly constitute cofactors for the enzyme, affecting the enzymatic activities required for physiological functions. Vitamin B complex is classified as B1 (Thiamine), B2 (Riboflavin), B3 (Niacin), B5 (Pantothenic acid), B6 (Pyridoxine), B7 (Biotin), B9 (Folate) and B12 (Cobalamin)^1,2^. Most of these vitamins cannot be endogenously synthesized and must be obtained through diet. Thiamine, Riboflavin, Niacin, Pyridoxine, and Biotin support energy metabolism during physical activity and rest periods. Meanwhile, Cobalamin is crucial in red blood cell production, tissue repair, and protein synthesis^3^. Additionally, Vitamin B12 and Folate are essential for fetal growth and development, particularly in the development of the infant brain. Pregnant and breastfeeding women particularly need to monitor their intake of Vitamin B6, as it plays a pivotal role in facilitating neuronal signals within the nervous system^4^. Clinically vitamin deficiency is quite common in infants and the elderly, therefore early treatment is essential to limit the damage and complications caused due to deficiency of vitamin. There are various methods for vitamin B detection such as Spectrophotometry^5^, Spectrofluorimetry^6^, Immunoassay^7^, HPLC (High-Performance Liquid Chromatography), HPLC-Ultra–Violet (UV)spectroscopy/Fluorescence Detection (FLD)/capillary electrophoresis^8–10^ and LC–MS (Liquid chromatography-mass spectrometry)^11,12^ which are expensive, time-consuming and bulky; therefore, a need for rapid, low cost and more reliable detection methods is essential.

Due to the successful outcome of glucose-detecting meters, and pregnancy test kits, biosensor devices have a wide range of applications that are interposed between the biomolecules and the sensing element to provide measurable signals. Looking into the sensing aspect, numerous biosensors are fabricated based on the Biosensing mechanism. Only a few of them are useful and applicable to biomedical applications and analysis. Among them, Nano-structured metal oxides (NMOs) are of prime importance as they have been extensively explored to develop biosensors with higher sensitivity, lesser response time, greater stability, and suitability. They render an effective surface for the immobilization of biomolecules with high activity, better orientations, and confirmation leading to enhanced sensing. NMOs are fabricated with metals like Zinc, Magnesium, Zirconium, Titanium, and Tin which exhibit interesting morphology, non-toxicity, biocompatibility, and catalytic properties. Most of them are in the form of Nano-rods, Nanofibers, and thin films of Nano-thickness, incorporating these NMOs with conducting and semiconducting nanoparticles like gold, silver, carbon nanotubes (CNTs), and graphene enhances their sensing. These structures are capable of interfacing bio-recognition events with the transduction of electronic signals and hence find potential applications in miniaturized, novel bio-sensing devices. High surface-to-volume ratio, confinement of electrons and phonons, better adsorption capability, and high efficiency of catalysis are remarkable optical and electrical properties of NMOs. NMOs pave the way for the fabrication of biosensors with enhanced sensitivity, improved limits of detection, and low cost of production. Among various NMOs, Zinc oxide (ZnO) has been attracting much attention due to its unique properties such as a wide direct band-gap (~ 3.3 eV) coupled with unusually high free exciton binding energy (60 meV), visible range transmittance, high electrochemical stability, non-toxicity and abundance in nature, makes it a promising candidate for several semiconducting and optoelectronic applications^13,14^. ZnO is a favourable choice for bio-sensing applications owing to its enhanced electron mobility, excellent adsorption capability stemming from its high isoelectric point (~ 9.5), better film-forming capability, and better chemical stability, as it is corrosion resistant. ZnO in various configurations such as Nano-rods^15,16^, Nano-flakes^17^, Nano-flowers, and Nano-spheres^18,19^ have been extensively used in various sensing owing to their ease of synthesis, chemical stability, and biocompatibility. The examination of morphology, particle size, and distribution characteristics of ZnO nanostructures has revealed significant advantages, notably enhanced crystallinity coupled with reduced structural defects. Synthesizing ZnO nanostructures at lower temperatures further contributes to their favourable properties, including good electrical conductivity. These attributes collectively render ZnO nanostructures highly conducive to the development of sensor devices characterized by rapid response times, stability, and reliability. Looking into the deposition aspect there are various techniques for the deposition of a metal-oxide thin film such as sol–gel spin coating^20–22^, pulsed laser deposition^23^, RF magnetron sputtering^23,24^, spray pyrolysis^25^, chemical vapor deposition^26^. The sol–gel method is advantageous over all other techniques as they are easy to prepare and cost-effective and allow tuning of film thickness by varying the precursor concentration or viscosity of the solution, spin speed, and evaporation rates during pre and post-heat treatments^27–30^.

One of the fastest-moving and most exciting interfaces is the use of quantum dots (QDs) due to their unique optical properties like narrow emission peaks, broad excitation spectra, and higher luminescence efficiency^31^. In the interaction of QDs with Biological systems the surface coating of QDs plays an important role. Despite the benefits and the concerns as to their exposure to human health, increased research on QDs to try and understand the interaction in-vivo and in-vitro has increased the interest regarding the interaction and its applicability as the clinical tool^32,33^. Many researchers have reported that quantum dots are used as fluorophores for detecting various toxic metal ions and biomolecules their resistance to photobleaching makes them conducive for optically excited Biosensing. As a result, extensive research is still required to understand the potential of QDs as a tool in medicine, and knowledge of QD-Biological system interaction is inadequate.

In the present work, homogeneous films of ZnO are grown on glass substrates using the sol–gel process. To improve the sensitivity and stability of the sensor, we have introduced Quantum dots (QDs). Metal contacts deposited on ZnO thin film enable its use as a resistive sensor to detect VB6.

## Experimental section

### Materials and methods

MPA-coated CdTe QDs were synthesized and its stock solution was prepared by dissolving an appropriate amount of CdTe QDs into the deionized water and stored in the refrigerator. Zn (CH_3_COO) 2.2H_2_O (Zinc acetate dehydrate), MEA (Monoethanolamine), conc.H_2_SO_4_ (Sulphuric acid), K_2_Cr_2_O_7_ (Potassium dichromate) and Pyridoxine (vitamin B6 (VB6)) were purchased from Sigma-Aldrich Chemical Co., USA. CH_3_–O–C_2_H_5_ (2-methoxy ethanol), was purchased from Loba Chemie Pvt Ltd.

### Instruments and characterization

The crystallinity and the phase of the synthesized ZnO thin film were obtained by Rigaku Ultima IV X-ray diffractometer with Cu Kα radiation. The morphological characterization of ZnO thin films was carried out by Zeiss Sigma 300 FESEM. Absorption spectra were acquired at room temperature using JASCO-UV 3600 with a 1.0 cm quartz cell. Fluorescence measurements of solutions were recorded using a Jasco FP8300 fluorescence spectrometer with a Xenon lamp (450W) and 1.0 cm quartz cell. The particle size of CdTe QDs was measured by using transmission electron microscopy (TEM) (HRTEM: Jeol/Jem 2100). Electrodes were deposited by Thermal vapour deposition with a custom-made shadow mask. The electrical measurements of QDs/Vitamin B6/Vitamin B6 + QDs sensing by the ZnO thin film-based device were carried out by KEITHLEY 2636 B SMU along with a probe station. Voltage has been swept from 0 to 2 V considering the low-power applications. DC Probe Station-PM5, Agilent Device Analyzer B1500A with pulsed source 5 MHz for impedance measurements.

### Synthesis of ZnO thin films

**Fig. 1:**
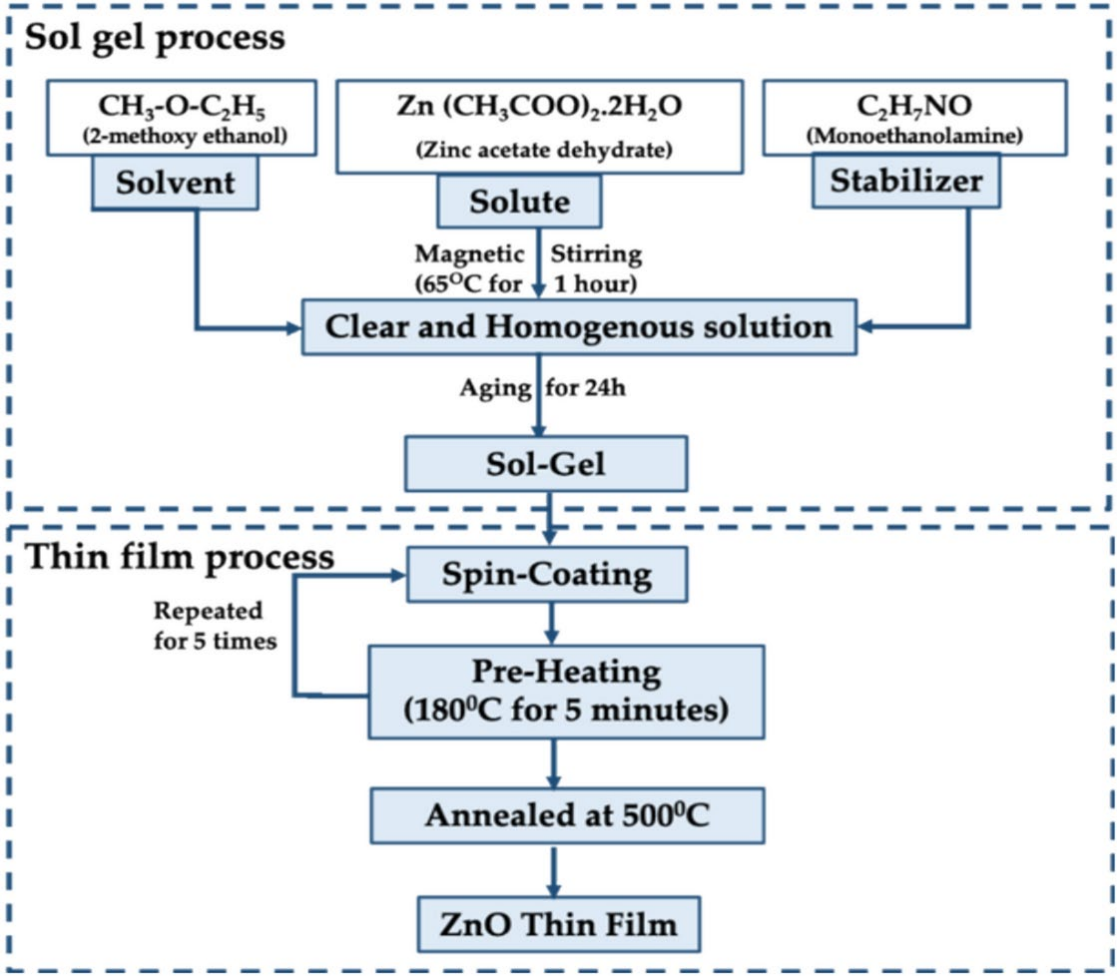
Flow chart of the thin-film deposition process.

The ZnO thin films were prepared using the sol–gel process as shown in the flow chart (Fig. 1). Initially, the glass substrates were dipped in chromic acid for 2 days to remove impurities, and the substrate surface was etched for better adsorption. The substrates were then rinsed with DI water and ultra-sonicated with isopropyl alcohol to remove any residual contaminants. A solution was prepared by dissolving zinc acetate dehydrate (ZAD) in a solution of 2-methoxy ethanol and Monoethanolamine (MEA). MEA was used as a soil stabilizer. The precursor concentration was fixed as 0.5 M and the molar ratio of ZAD to MEA was maintained at 1:1.

The mixture was placed on a hotplate and stirred at 250 rpm at a temperature of 65 for 1 h. The resultant clear, homogeneous solution was aged for about 24 hours^34^. The seed solution prepared was dropped onto pre-cleaned substrates and spun at a speed of 3000 rpm on a spin coater for a duration of 30. The slides were heated at 180 °C for 5 min after each coating. The procedure was repeated for 5 iterations. The coated ZnO thin film was then annealed at 500 °C in an air furnace for 1 h. To fabricate the proposed Vitamin B6 sensor, ZnO thin film was used as a sensing platform. The silver electrodes of width ~ 1 mm of channel gap with custom-made shadow mask were deposited by thermal vapour deposition technique and the final sensing platform was obtained.

### Synthesis of CdTe quantum dots

Water-soluble CdTe QDs by using dual capping agents namely MPA and Citric acid have been synthesized via a one-pot synthesis^35^.CdCl_2_, K_2_TeO_3_, NaBH_4,_ and citric acid were serially added in the molar ratio of 1:0.2:3.2:5.2 in 30 mL of distilled water, maintaining pH 9 by adding sodium hydroxide in the presence of MPA as stabilizing agent. The resulting solution was then subjected to heating for 2 h at 80 °C to promote the growth of QDs. The precipitate was washed several times and resuspended in DI water.

### Preparation of samples

The VB6, and Cadmium Telluride (CdTe) quantum dots (QDs) samples are as follows. 1 mg of VB6 is dissolved in 20 ml water to obtain a stock solution. The sample 1 comprises aqueous CdTe QDs at a constant concentration. Sample 2 comprises VB6 at a varying concentration ranging from 2-10uM. The total volume of sample 1 and sample 2 is maintained at 2 ml by adding CdTe QDs.

## Results and discussion

### XRD analysis for structural characterization

**Fig. 2:**
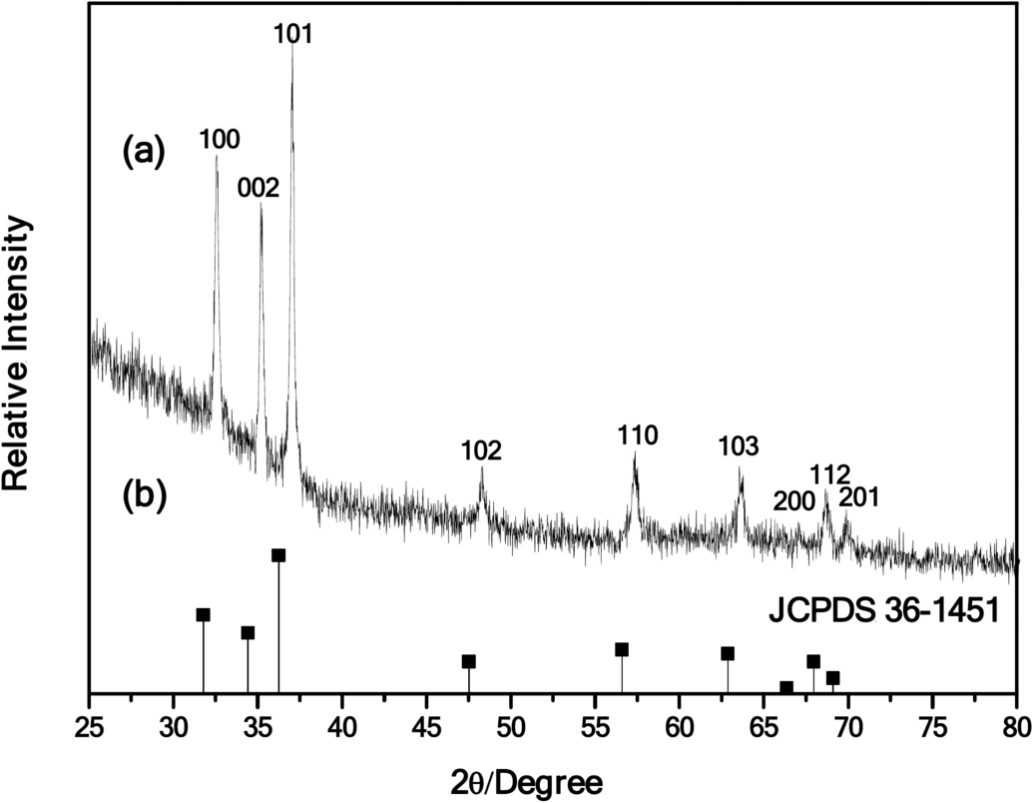
XRD spectra of ZnO thin film grown using sol–gel on glass substrate annealed at 500 °C matching with JCPDS 36-1451.

Figure 2 represents the diffraction pattern of the ZnO thin film, and the pattern shows several peaks corresponding to the (100), (002), (101), (102), (110), (103), (200), (112) and (201) planes of standard polycrystalline hexagonal ZnO. The X-ray diffraction (XRD) patterns of the ZnO thin films closely match those specified in the JCPDS card 361451. The narrow diffraction peaks indicate the good crystalline structure formation and the mean crystallite size was ~ 40 nm calculated from the Debye Scherer formula. The XRD pattern doesn’t show any other peaks other than the ZnO peak, which indicates that the obtained thin film is free of impurities.

### Morphological characterization

**Fig. 3:**
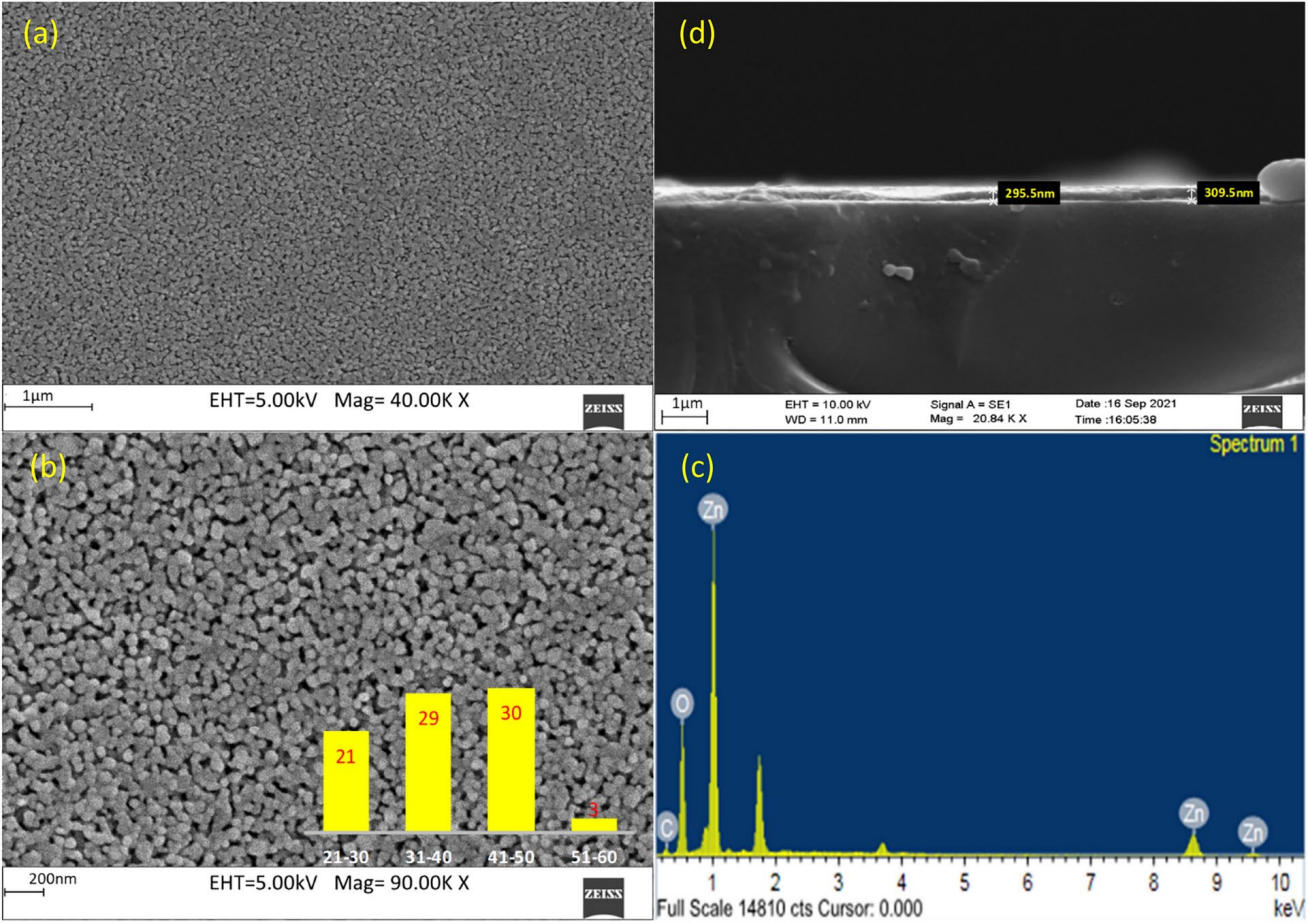
FESEM images of ZnO thin film grown by sol–gel method with precursor ratio of 1:1 onto a glass substrate kept at 500 °C using 5 deposition cycles, depicted in images (**a**), (**b**), and (**c**) respectively. The linear dimensions are in the range of (**a**) 1 µm (**b**) 200 nm respectively (Insert figure shows size distribution) & (**c**) EDS spectra (**d**) cross-sectional view.

The surface properties of the film play an important role in the electrical properties of the device. Figure 3a,b indicates FESEM images of ZnO thin film grown by sol–gel method with a precursor ratio of 1:1 onto a glass substrate annealed at 500 °C. The film was uniform as seen by the film coverage and the size distribution shows the average particle size of ~ 40nm. Intense peaks shown in the EDX spectra referred to zinc and oxygen (Fig. 3c). Figure 3d shows the FESEM images of a cross-sectional view of the film depicting a thickness of around ~ 300 nm.

### Characterization of CdTe quantum dots

**Fig. 4:**
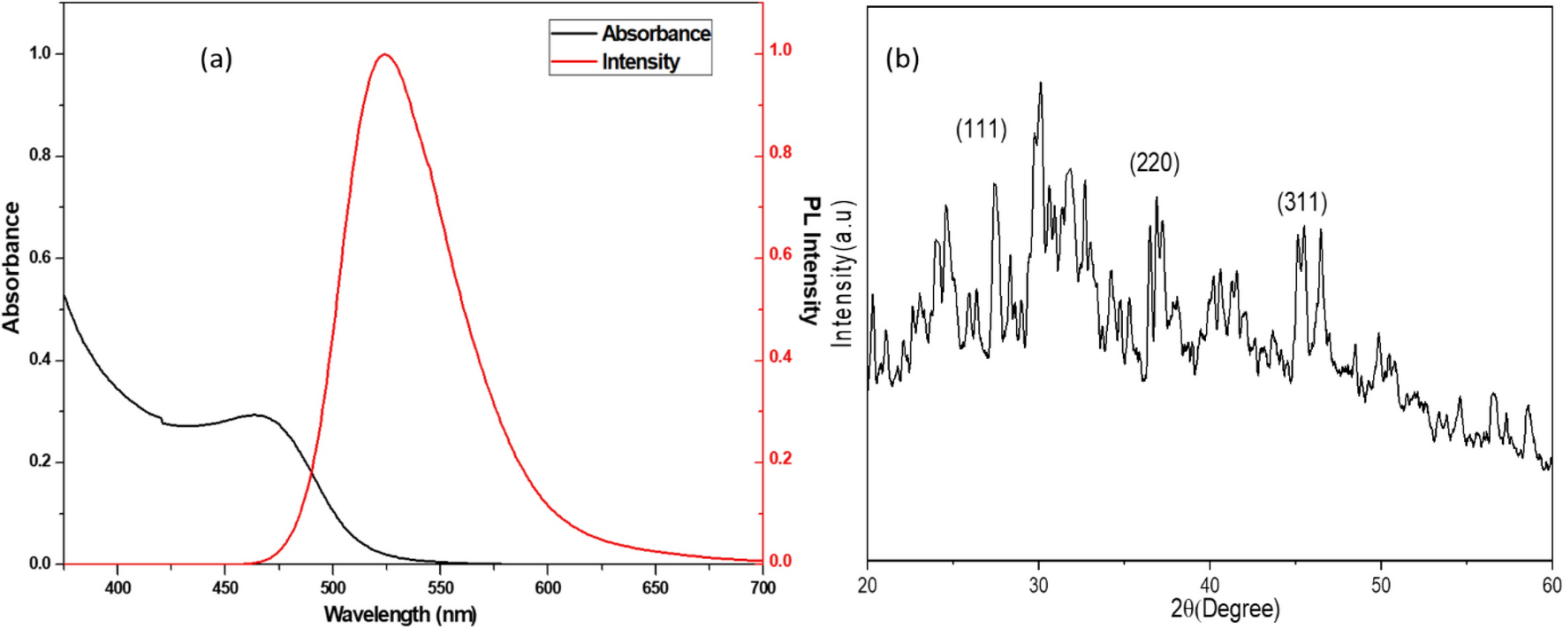
(**a**) Absprtion and emmsion of synethsied QDs (**b**) XRD spectra.

The synthesized CdTe QDs showed broad absorption characteristics with narrow emission spectra at emission maxima at 525 nm (Fig. 4a Absorption and emission of synthesized QDs and 4(b) show the XRD spectra) which is tunable to any desired spectral wavelength in the UV–visible region. The synthesised QDs are highly luminescent and are highly stable in water with large negative Zeta potential. The size of the synthesized CdTe QDs, approximately 3 nm, was calculated using the equation reported by Peng et al.^40^, where ‘D’ (nm) represents the size of the CdTe QDs, and ‘λ’ (nm) is the wavelength of the first excitonic absorption peak of the CdTe QDs.

$${\text{D}} = \left( {9.8127 \times 10^{ - 7} } \right)\uplambda ^{3} - \left( {1.7147 \times 10^{ - 3} } \right)\uplambda ^{2} + \left( {1.0064} \right)\uplambda - \left( {194.84} \right)$$

**Fig. 5:**
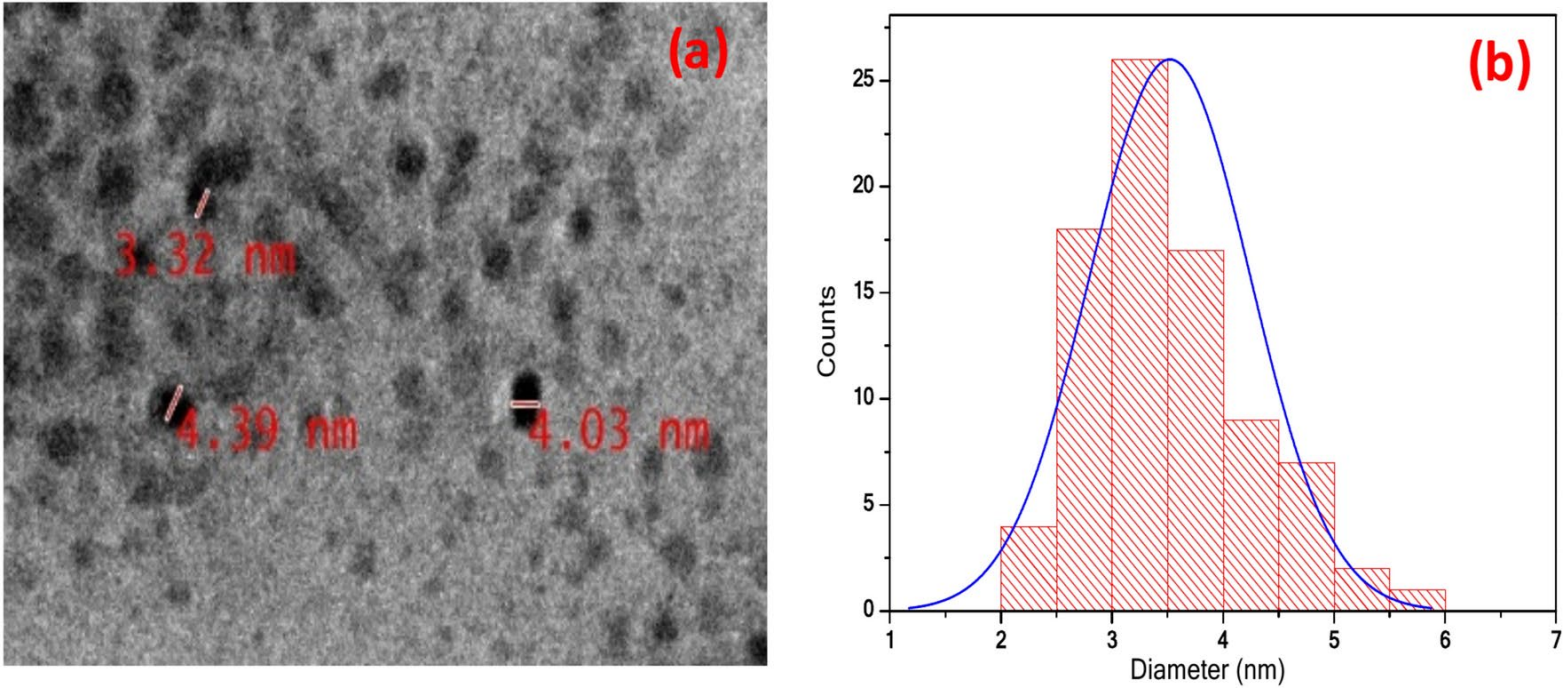
(**a**) TEM micrographs of CdTe QDs (**b**) size distribution.

For the confirmation of the size of the QDs, a TEM image was obtained (Fig. 5a), which shows the average diameter was ~ 3.5 nm and particle distribution was ranging on average from 2 to 6 nm (Fig. 5b).

### I–V characteristics measurements

**Fig. 6:**
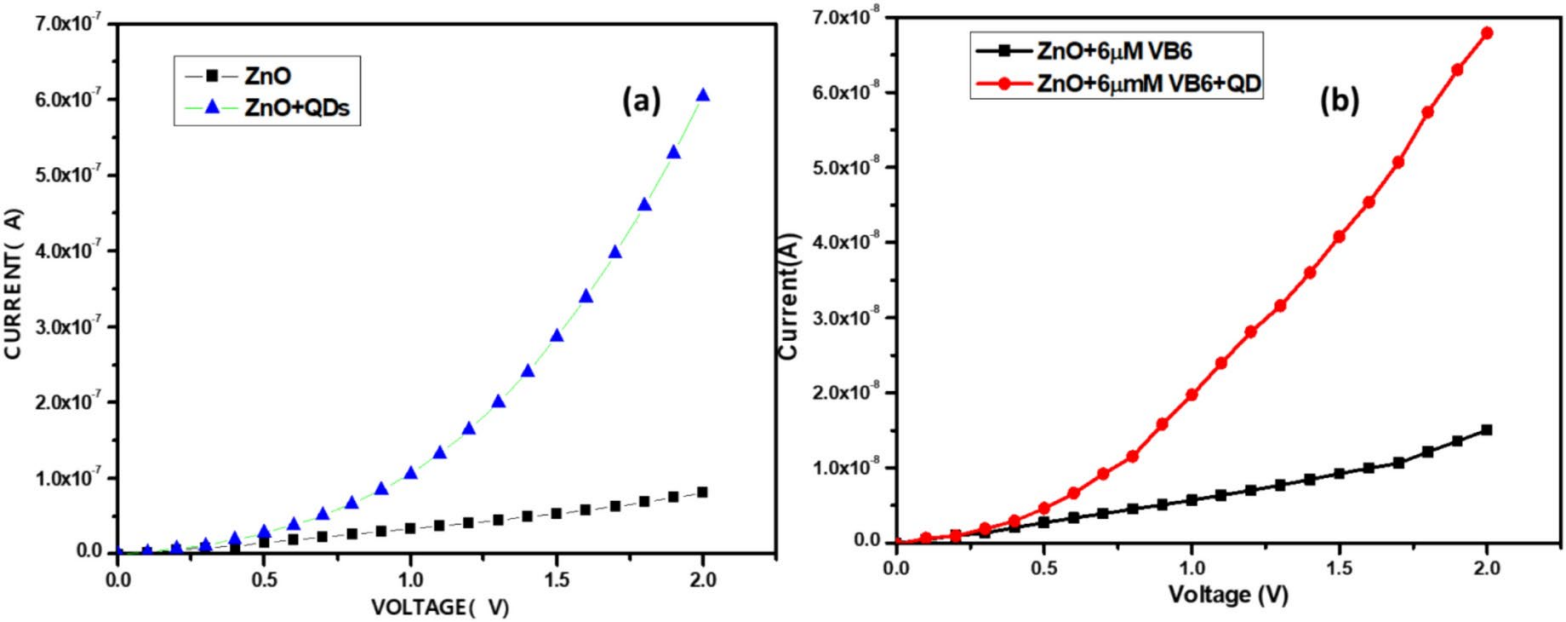
Current versus voltage (I–V) characteristic (**a**) before and after the addition of QD on the ZnO (**b**) before and after the addition of QD on the ZnO in the presence of VB-6.

Initially, current versus voltage (I–V) measurements have been measured for the desired voltage range starting with ZnO thin film alone and ZnO thin film + CdTe QDs without any VB6. Figure 6a shows the I–V measurements of ZnO and ZnO along with CdTe QDs. Observations revealed that the bare ZnO thin film exhibited minimal to negligible current, as illustrated in Fig. 6a. Based on the result, the current responses have shown significant changes when the developed sensor was treated with CdTe QDs on ZnO as in Fig. 6a.

**Fig. 7:**
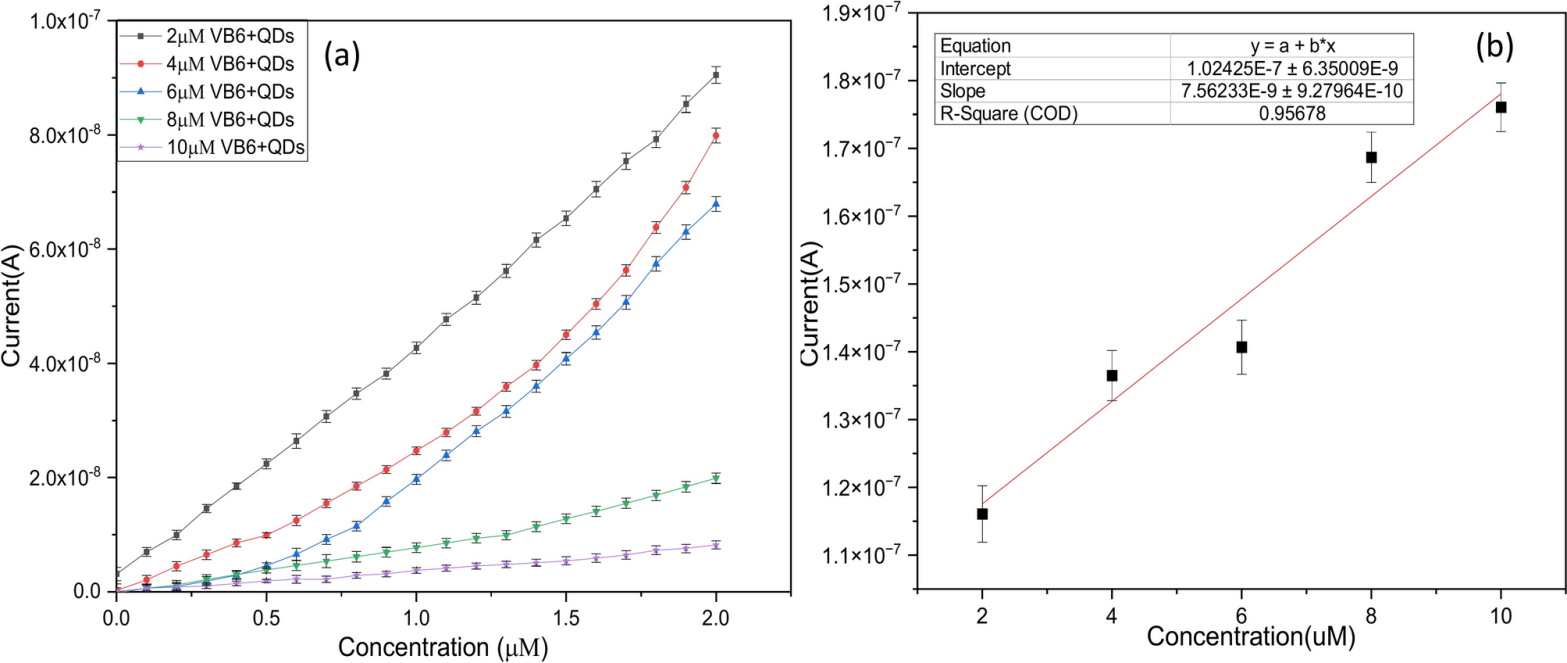
(**a**) I–V characterization of ZnO surface after adding a known concentration of QDs to VB6 (2–10 µm), with error bars representing the standard error (n = 5). (**b**) Sensor calibration curve, where error bars indicate the standard error from five measurements.

Similarly, the experiment was carried out to understand the response of Vitamin B6 on the ZnO thin film. Where a drop (0.5μL) of the solution is drop cast between the electrode and the corresponding current is measured after drying the solution at 55 °C. It was observed as in Fig. 6b VB6 has a lower current response when compared to that with the combination of VB6 + QDs on the ZnO surface. Therefore, to enhance the signal, we used quantum dots of fixed concentration for the detection of vitamin B6. Figure 7a shows the electrical characterization conducted on the surface of ZnO after the addition of various concentrations of Vitamin B6 for a fixed concentration of QDs. Based on the current–voltage relationship, it can be deduced that the device demonstrated a linear correlation between the current and voltage.

The current versus voltage (I–V) onto the ZnO thin film for various concentrations, showed a decrease in current as the concentration increased (Fig. 7a). The error bars reported in the figures are for five independent repeats (n = 5), ranging from 10^−9^ to 10^−10^ (0.1–1 nA).

To understand the amount of interference of VB6 alone under the presence of CdTe QDs calibration plot was plotted where the output signal (current): CdTe QD-(CdTe QD + VB6) measured as a function of VB6 concentration with a fixed bias 1.5 V is shown in Fig. 7b (Showing the contribution of VB6 alone for 1.5 V) along with the standard error bars for five trails. From the current-to-voltage relationship, it can be inferred that the device exhibited a linear relation between the current and voltage. Furthermore, parameters like sensitivity and lower limit of detection are very essential for sensor performance. The sensitivity and the lower limit of detection of the fabricated device were calculated from the linear range plot that starts from 2 to 10 µM for a fixed bias of 1.5 V. The sensitivity of the sensor is defined as the slope of the output characteristic curve (Fig. 7b). The obtained sensitivity of the fabricated device was 7.56 ± 0.99 nA/µM and the lower limit of detection and limit of quantification was 0.9 µM and 2.7 µM (*R*^2^ = 0.96, n = 5)(Showing the contribution of VB6 alone for 1.5 V) .

## Electrochemical impedance spectroscopy (EIS) study

To comprehend the kinetics of charge transfer in metal oxide thin films, electrochemical impedance spectroscopy, or EIS, is a helpful technique. To fully examine the capacitive properties and the electron transport behaviours, an EIS study was performed. The impedance characterization of the VB6, QDs & VB6 + QDs was done using sinusoidal alternating current (ac) supplied between the two Ag electrodes in the frequency range from 1 to 100 kHz for measurements of impedance spectra by a DC Probe Station-PM5, Agilent Device Analyzer B1500A with pulsed source 5 MHz respectively. The experiment was conducted at 1.5 V. The electrochemical impedance spectroscopy (EIS) measurements for the VB6/ZnO/glass, VB6 + QDs/ZnO/glass, and QDs/ZnO/glass were conducted to evaluate the effectiveness of the sensor fabrication process. EIS is a widely utilized technique for examining changes in surface states and charge transfer properties. The obtained EIS data were modelled using a Randles equivalent circuit, which includes parameters such as solution resistance (Rs) in series with C_1_ corresponding to the capacitance of the coating whilst R_2_ corresponding to the resistance of the metal interface and a constant phase element (CPE) in parallel representing the double-layer capacitance.

**Fig. 8:**
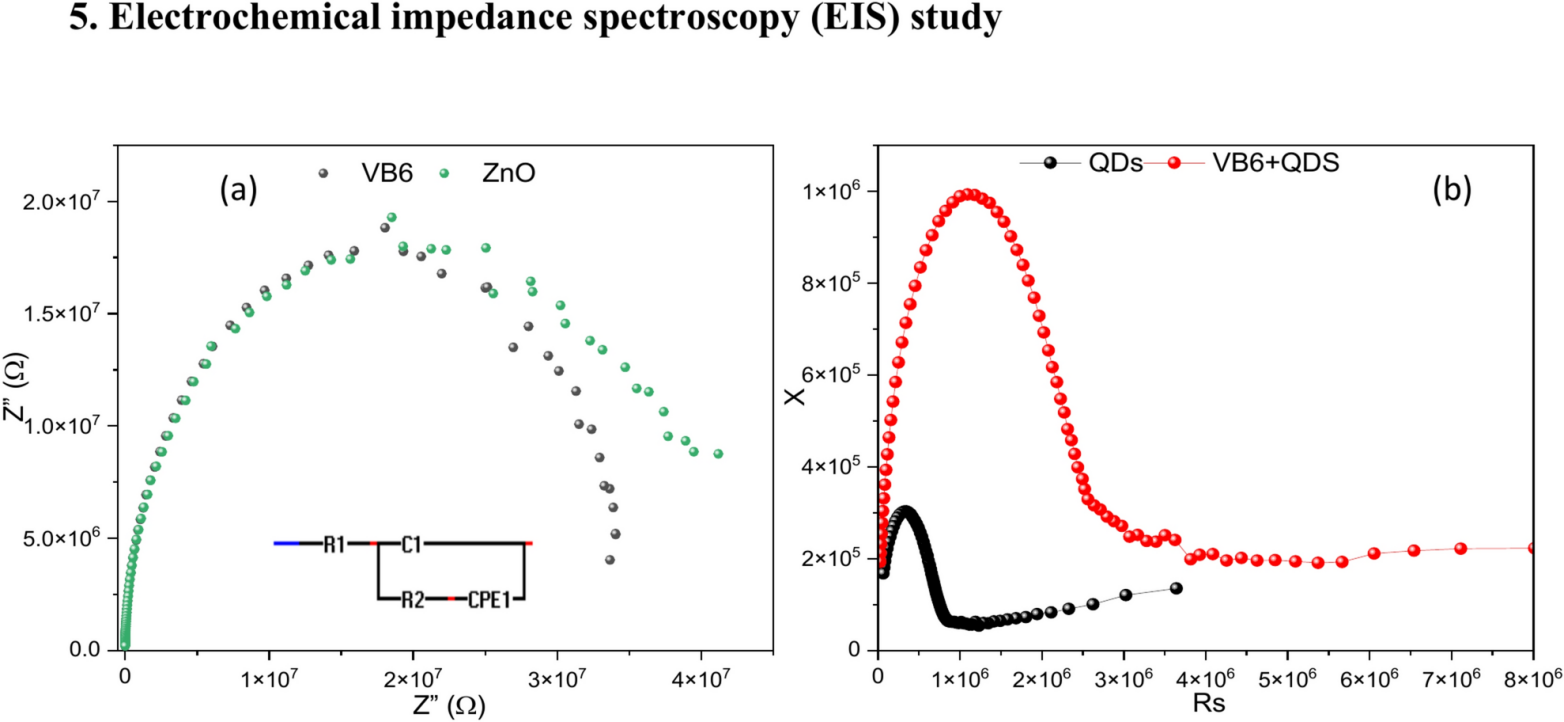
Typical Nyquist plots of the imaginary part of the impedance (Z′) versus the real part of the impedance (Z″) for (**a**) VB6 & ZnO (**b**) QDs, and VB6 + QDs within a frequency range from 1 kHz to 1 MHz with applied amplitude of ± 1.5 V (insert figure showing the equivalent circuit).

The Nyquist plot’s semicircle diameter corresponds to the charge transfer resistance (Rct), reflecting the charge transfer kinetics at the surface. As depicted in the corresponding Fig. 8, the EIS spectrum for the ZnO/glass electrode 8(a) displayed a lower Rct value, indicative of a superior charge transfer rate attributed to the high conductivity of the QDs(8(b)). Conversely, the Nyquist semicircle for the VB6 + QDs/ZnO/glass electrode 8(b) is larger, suggesting reduced conductivity due to the presence of VB6, which impedes electron transfer. For the VB6/ZnO/glass electrode(8(a)), the Rct value increased further, likely due to the non-conductive nature of VB6, which significantly obstructs electron transfer similar to ZnO/glass. The result obtained here is similar to the current response result in the I–V.

Incorporating QDs improves the charge transfer properties due to their high conductivity, whereas adding VB6 decreases conductivity and hinders electron transfer. Therefore, due to its superior charge transfer characteristics, the QDs/ZnO/glass demonstrates the most effective fabrication process for enhancing sensor performance.

**Fig. 9:**
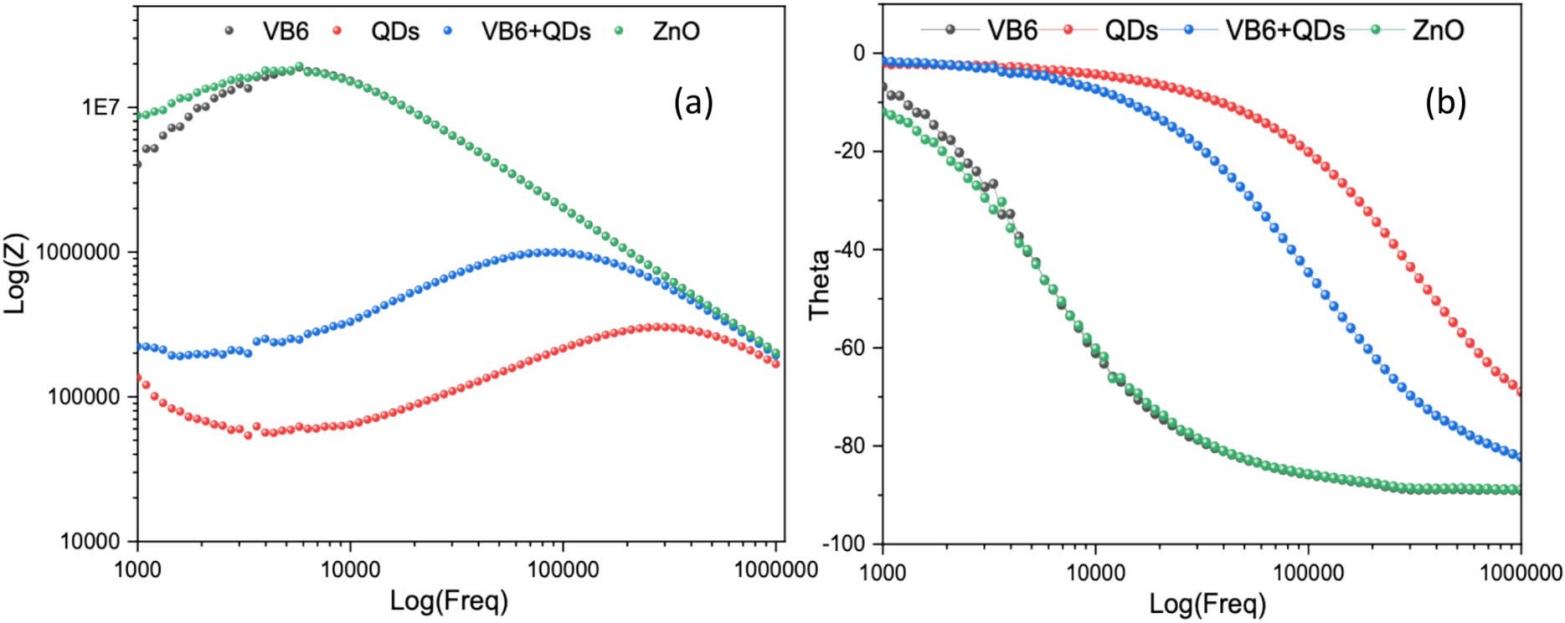
(**a**) Bode diagrams (module |Z| vs. frequency) (**b**) phase plot.

Figure 9a represents the bode plots for VB6, QDs, VB6 + QDs & ZnO showing that the combination of VB6 and QDs frequency shift from the natural frequency (ZnO, VB6). Each material has unique magnitude and phase responses as shown in Fig. 9b indicating different interactions with the frequency range tested. The combination of VB6 and QDs shows different magnitude and phase characteristics suggesting possible interactions. The peaks and phase response shifts and modifications shed light on the interactions and frequency-dependent behaviour of these materials. Based on the phase plot the lifetime of Charges (relaxation time) is calculated and shown in the table below. The variations in charge carrier lifetimes between ZnO, VB6, QDs, and VB6 + QDs as seen.

ZnO and VB6 have similar lifetimes, which implies that in a device, their charge carrier recombination kinetics can be similar (Table 1). This may suggest that ZnO and VB6 have comparable roles in the processes of charge production and recombination inside the device structure. Compared to ZnO and VB6, QDs have a shorter lifespan, which suggests faster recombination or loss of charge carriers. This property could indicate that QDs are not as effective in maintaining or moving charges around the device.

**Table 1:** The variations in charge carrier lifetimes between ZnO, VB6, QDs, and VB6 + QDs.

Materials	Life-time of charges (s)
ZnO	2.76544E−05
VB6	2.76544E−05
QDs	4.80578E−07
VB6 + QDs	1.59134E−06

## Selectivity and anti-interference ability studies

**Fig. 10:**
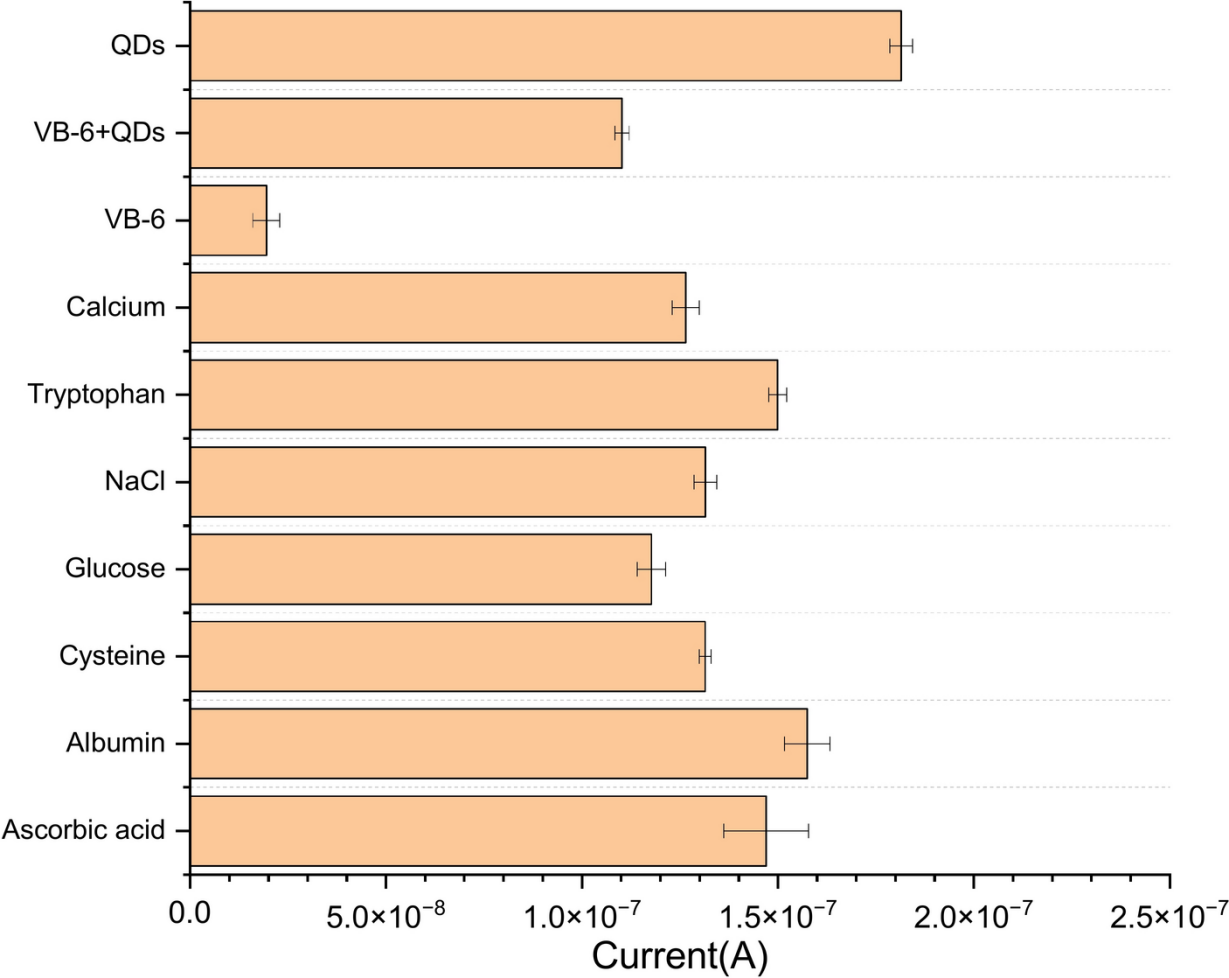
Interference study of the sensor; the responses of AA, BSA, cysteine, glucose, NaCl, tryptophan and calcium in the presence and absence of 4.0 µM Vitamin + QDs at the fabricated sensor, applied working potential of 1.50 V (histogram of the current response).

To assess the selectivity of the sensor, various common interferents, including AA, BSA, Cysteine, Glucose, NaCl, Tryptophan and Calcium, were individually introduced into test solutions. The concentrations of these interfering substances were set higher (around 5 mM) than the concentration of VB-6 (4 μM). Figure 10 illustrates the sensor’s response, revealing a robust sensitivity to VB-6 in comparison to other Interference ions. Furthermore, to evaluate the impact of interfering substances on the detection of VB-6 the sensor was exposed to a mixture of VB-6 + QDs + each of the interfering ions. The results demonstrated that the sensor maintained its strong responsiveness to VB-6 even in the presence of these interfering substances. The sensor exhibited excellent selectivity and anti-interference capabilities owing to the specific coordination between VB6 and QDs. This underscores the sensor’s ability to selectively detect VB-6 amidst a range of potential interferents, highlighting its practical utility in applications requiring accurate and specific detection of VB-6^36^.

**Fig. 11:**
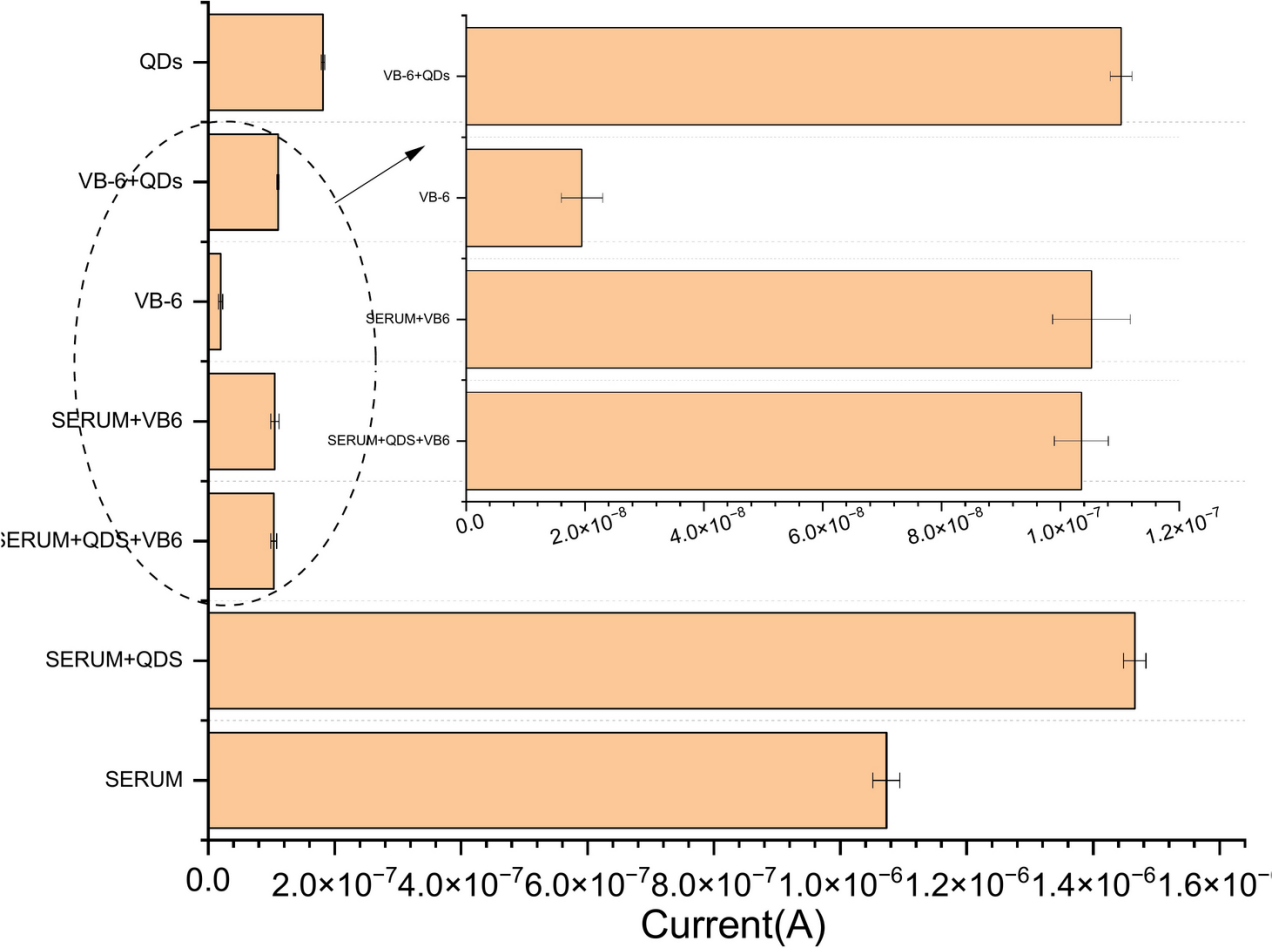
The responses of serum in the presence of VB-6 + QDs applied a working potential of 1.50 V (error bars indicate standard error for n = 5).

For analytical applications, the proposed sensor was tested for VB6 detection in serum samples (Fig. 11). the percent recovery values for VB6 determination by our electrode are 106.4%, and the R.S.D. values are found to be 4.35% less than 5%. This suggests that the proposed sensing platform offers good selectivity and repeatability for the determination of VB6 in a real sample matrix (human serum).

## Sensor device performance

The principle of operation of the sensing device depends on varying resistance of the zinc oxide thin-film with different concentration of Pyridoxine mediated by CdTe QDs. ZnO having a high isoelectric point of about 9.5 shows that a huge number of positive charges (Zn^2+^) exits on its surface^37^. It can be noted that the sensor showed a low response in the absence of CdTe. Once the analyte solution is placed on the sensing platform, Pyridoxine gets adsorbed onto the ZnO surface. Now the addition of a known concentration of CdTe with the pyridoxine molecules gets adsorbed upon the ZnO thin-film surface due to electrostatic attraction between CdTe and ZnO resulting in the increase in current. The charge transfer transition may happen while the transition metal ion Zn^2+^ binds with CdTe^37,38^ Owing to the ionization of Zn atoms, plenty of electrons are available in ZnO conduction so that it becomes an n-type semiconductor. However, the conductivity of ZnO may alter due to the adsorption of some molecules on its surface^39,40^. A larger concentration of pyridoxine molecules reduces the adsorption result the less no of electrons. As a result, the device current decreases with the increment of Pyridoxine concentration. It is also seen that variation with the increment of pyridoxine concentrations mediated by CdTe while the applied biases were kept at a fixed value^41^. The mechanism of the adsorption kinetics of biomolecules on solid/liquid surfaces has been explained by several research groups^42^. Usually, the adsorption on the ZnO surface is through the COOH anchoring group of CdTe. One hydrogen atom migrates from the COOH group of the CdTe QDs to the oxygen vacancy sites of ZnO whenever the adsorption occurs through the COOH functional group. Adsorption of pyridoxine occurs through the –NH group with the binding of nitrogen atom to a surface Zn atom. The surface energy of ZnO undergoes alteration upon the adsorption of molecules, as evidenced by the variation in current observed when changing the concentration of pyridoxine. However, as the interaction between B6 and B6 increases, the rate of electron transfer slows down. Now, the current through the ZnO after the adsorption of B6 increases rapidly with respect to the bare ZnO. On addition of CdTe solution, there is a maximum increase of current which indicates the adsorption on the surface by the COOH group of the CdTe QDs to the oxygen vacancy sites of ZnO. The significant impact of B6 adsorption is observed through the interaction with ZnO, resulting in a rapid enhancement of electron transfer rates.

In comparison to other methods, the number of published reports on the determination of vitamin B6 is limited. Table 2 provides a summary of various sensing methods for vitamin B6 detection. Typically, these methods utilize expensive materials, and the operational procedures appear to be complex before analysis. However, our QDs-based sensing approach for vitamin B6 offers several advantages. Firstly, it is easier to fabricate compared to previously published methods. Additionally, our method requires a low volume of analyte (0.5 µL) for detection. Therefore, this proposed method represents a superior system for determining vitamin B6 compared to other published methods. Furthermore, it can find utility in pharmaceutical companies for analyzing vitamin B6 content in tablets and injections, as well as in real-time sample matrices.

**Table 2:** Summary of various sensing methods for vitamin B6 detection.

Sensor	Technique	Linear range	LOD	Reference
PEDOT/Fc^−^/GCE	CV	0.1–300 mM	0.05 mM	^43^
		0.5–1500 mM	0.1 mM	
		1.5–2000 mM	0.7 mM	
Silver-doped poly (l-arginine)-GCE	CV	1.0 × 10^–5^–3.0 × 10^–3^ M	5 × 10^–6^ M	^44^
Au-CuO/MWCNTs/GC-modified electrode	CV	0.79 mM–18.4 mM	0.15 mM	^45^
MWCNT-modified screen-printed electrode	DPV	2.0 × 10^−6^–7.2 × 10^−5^ M	1.5 × 10-^6^ M	^46^
PEDOT/ZrO_2_NPs/GCE	DPV	0.5–1000 μM	0.20 μM	^47^
GC/MWCNTs-Mn^III^salen	DPV	1.0–300 μM	0.42 μM	^48^
F-MWCNTs modified GCE	CV	0.5 to 20 µM 20 to 200 µM	0.038 µM	^49^
	EIS		0.125 µM	
Pencil graphite electrode modified with gold nanoparticles	CV	5 to 200 μM	0.30 μM	^50^
Carbon paste (CP) with iron oxide nanoparticles (IONs)	EIS, CV, DPV	8.88–1000.0 µM	9.06 µM	^51^
CdTe QDs	Optical	33.22–445.9 mg/mL	0.15 mg/mL	^52^
CdTe QDs-ZnO thinfilm	I–V	2–10 µM	0.906 µM	This work

## Conclusion

This study demonstrates the successful development of a ZnO thin-film-based biosensor enhanced with CdTe quantum dots for the detection of Vitamin B6 (Pyridoxine). The sensor, fabricated using the spin-coating sol–gel technique and annealed at 500 °C, exhibited a high sensitivity of 7.56 ± 0.92nA/µM and a detection limit of µM (n = 5), enabling reliable detection in the range of 2–10 µM. The integration of CdTe QDs facilitated efficient charge transfer, enhancing the sensors performance while operating at a low voltage (< 2 V). The sensing mechanism is primarily governed by charge transfer interactions, specifically electrostatic attraction between Vitamin B6 (mediated by CdTe QDs) and the ZnO thin film. Despite its advantages, challenges such as potential sensor degradation over prolonged use and matrix interference in complex biological samples need further investigation. Future improvements could focus on enhancing sensor stability, optimizing surface functionalization for improved selectivity, and exploring miniaturization for portable and real-time applications. With its simple fabrication, low analyte volume requirement, and cost-effectiveness, this ZnO-CdTe QD-based sensor presents a promising alternative to conventional Vitamin B6 detection methods. It holds significant potential for applications in pharmaceutical quality control, point-of-care diagnostics, and real-time biomedical analysis.

## Data Availability

Data sets generated during the current study are available from the corresponding author on reasonable request.
